# Gender Differences in Transcriptional Signature of Developing Rat Testes and Ovaries following Embryonic Exposure to 2,3,7,8-TCDD

**DOI:** 10.1371/journal.pone.0040306

**Published:** 2012-07-09

**Authors:** Solange Magre, Diane Rebourcet, Muhammad Ishaq, Richard Wargnier, Cyrille Debard, Emmanuelle Meugnier, Hubert Vidal, Joëlle Cohen-Tannoudji, Brigitte Le Magueresse-Battistoni

**Affiliations:** 1 Université Paris Diderot, Sorbonne Paris Cité, Biologie Fonctionnelle et Adaptative, EAC CNRS 4413, Paris, France; 2 Université Lyon 1, INSERM U1060, INRA U1235, CarMeN, Laboratoire Lyonnais de Recherche en Cardiovasculaire, Métabolisme, Diabétologie et Nutrition, Oullins, France; University of Maryland School of Medicine, United States of America

## Abstract

Dioxins are persistent organic pollutants interfering with endocrine systems and causing reproductive and developmental disorders. The objective of our project was to determine the impact of an *in utero* exposure to 2,3,7,8-tetrachlorodibenzo-p-dioxin (TCDD) on reproductive function of male and female offspring in the rat with a special emphasis on the immature period. We used a low dose of TCDD (unique exposure by oral gavage of 200 ng/kg at 15.5 days of gestation) in order to mirror a response to an environmental dose of TCDD not altering fertility of the progeny. We choose a global gene expression approach using Affymetrix microarray analysis, and testes of 5 days and ovaries of 14 days of age. Less than 1% of the expressed genes in gonads were altered following embryonic TCDD exposure; specifically, 113 genes in ovaries and 56 in testes with 7 genes common to both sex gonads. It included the repressor of the aryl hydrocarbon receptor (Ahrr), the chemokines Ccl5 and Cxcl4 previously shown to be regulated by dioxin in testis, Pgds2/Hpgds and 3 others uncharacterized. To validate and extend the microarray data we realized real-time PCR on gonads at various developmental periods of interest (from 3 to 25 days for ovaries, from 5 to the adult age for testes). Overall, our results evidenced that both sex gonads responded differently to TCDD exposure. For example, we observed induction of the canonic battery of TCDD-induced genes coding enzymes of the detoxifying machinery in ovaries aged of 3–14 days of age (except Cyp1a1 induced at 3–10 days) but not in testes of 5 days (except Ahrr). We also illustrated that inflammatory pathway is one pathway activated by TCDD in gonads. Finally, we identified several new genes targeted by TCDD including Fgf13 in testis and one gene, Ptgds2/Hpgds regulated in the two sex gonads.

## Introduction

Dioxins, which refer to a family of structurally and chemically related polychlororinated dibenzo-*p*-dioxins (PCDDs) and dibenzofurans (PCDFs) are lipophilic chemicals resistant to degradation and categorized as persistent organic pollutants, with 2,3,7,8- tetrachlorodibenzo-p-dioxin (TCDD) being the most toxic dioxin. Dioxins are suspected of interfering with the endocrine systems of humans and wildlife [1]–[3] causing a broad spectrum of adverse effects including developmental and reproductive toxicity in the offspring of laboratory animals, and perhaps in humans. These disorders are of very high concern, because they occur at much lower doses than those causing wasting syndrome or carcinogenesis [4]. Moreover, dioxins tend to accumulate in the food chain, essentially fatty food including breast milk, and may also cross the placental barrier. These data emphasize that foetuses and neonates are vulnerable populations. For example, it was recently demonstrated that breast-fed but not formula-fed sons from mothers exposed to dioxin after the accident in Seveso had permanently reduced sperm quality [5]. In utero exposure to TCDD also impaired prostate development in many mammals including rats and mice. However, data on testicular development are debated [6]–[8]. In rat females, it has also been shown that TCDD exposure in utero is associated with malformations of external genitalia, reduced fertility, and disruption of estrus cycles and inhibition of ovulation [9], [10].

**Table 1:** List of primers used in the study.

List of primers	Accession number	Forward 5'-3'	Reverse 5'-3'	size (bp)
Ahrr	NM_001024285.1	CAGCAACATGGCTTCTTTCA	GAAGCACTGCATTCCAGACA	172
Cyp1a1	NM_012540.2	CAAGAGCTGCTCAGCATAGTC	GCTCAATGAGGCTGTCTGTG	229
Cyp1b1	NM_012940.1	GCAGCCGCCTTCCTGGTAGC	CCACGCGCCCTGTCCCTACT	116
Nqo1	NM_017000.3	TGCTTTCAGTTTTCGCCTTT	GAGGCCCCTAATCTGACCTC	122
Cyp19a1	NM_017085.2	TGTTGTTGGTGACAGAGACA AAG	CAAGTCCACGACAGGCTGATA	103
Star	NM_031558.2	GTCATCAGAGCTGAACACGG	TGGCGAACTCTATCTGGGTC	163
Art2b	NM_198735.2	TGTGGTTCTCCCCAGTCTTC	CTCCTTGGCCTCCCTTTAAC	104
Fgf13	NM_053428.1	TGTATCGTCAACCCCAGTCA	GCCACTGTTCCACAGTTCCT	139
Gzmf	NM_153466.1	CAAATGTCCGTCGATGTCAC	CCCTTGTACGCAGCCTGTAT	158
Hpgds	NM_031644.2	AGAGCGGACGTTCAATGACT	GGTGCTGCAGATATCCCAAT	139
Hprt1	NM_012583.2	AGGACCTCTCGAAGTGT	ATTCAAATCCCTGAAGTACTCAT	111

Most of the toxic effects of TCDD are mediated through the binding and activation of the aryl hydrocarbon receptor (AHR) and subsequent alteration of target gene expression. The classic battery of dioxin-responsive genes exhibit dioxin response elements in their promoter moiety and includes phase I and phase II enzymes of the detoxification machinery, such as the cytochrome P450 (Cyps) 1a1 and 1b1. However, a recent study pointed out that 65% of the gene expression responses elicited by TCDD do not involve direct AhR binding to a Xenobiotic Response Element (XRE) [11]. It suggested that expression of genes lacking a XRE element reflected indirect AhR-mediated signalling, and that such changes in expression could be attributed to latent secondary effects. It could as well suggest that non-consensus XRE element may confer TCDD inducibility as shown recently with PAI-1 [12]. These two mechanisms may well explain the pleiotropic nature of TCDD toxicity illustrated with the large panel of target genes identified that include for example genes involved in cellular growth, differentiation and inflammation [13], [14].

**Figure 1:**
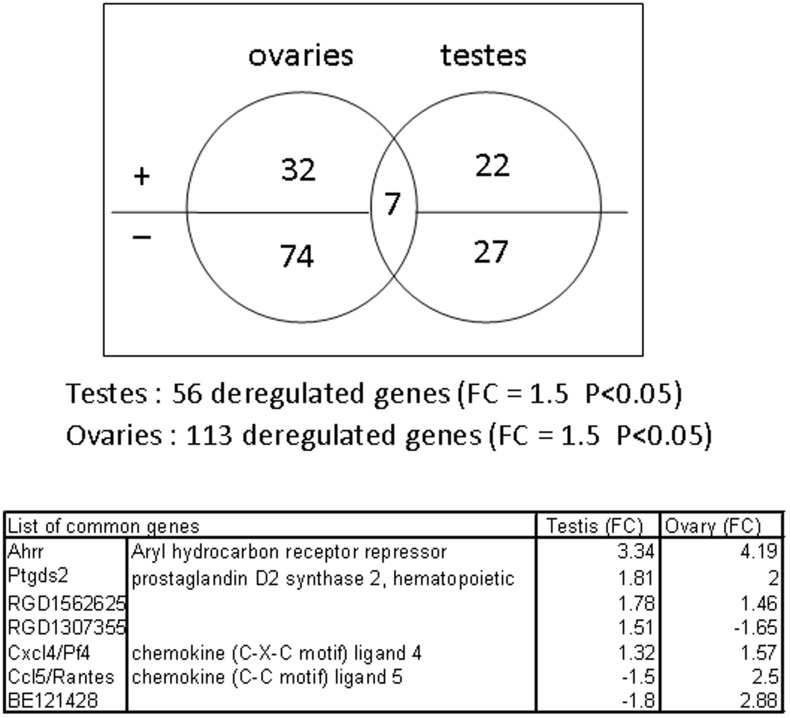
TCDD-regulated genes detected by microarrays in ovaries and testes. Genes were selected on the basis of a *p-*value<0.05 and a 1.5 fold change factor of mRNA expression in response to TCDD. The Venn diagram illustrates the repartition between genes up- and down-regulated in testes or ovaries, and the 7 genes both regulated in testes and ovaries are listed.

**Table 2:** Sub-list of relevant genes TCDD-regulated in testes.

Gene Title	Gene Symbol	Fold Change
ADP-ribosyltransferase 2b	Art2b	8.23
granzyme F	Gzmf	4.40
aryl-hydrocarbon receptor repressor	Ahrr	3.34
ficolin B	Fcnb	2.00
peroxisomal biogenesis factor 11 alpha	Pex11a	1.87
prostaglandin D2 synthase 2, hematopoietic	Ptgds2/Hpgds	1.81
protein kinase (cAMP-dependent, catalytic) inhibitor beta	Pkib	1.78
versican	Vcan	1.68
fibroblast growth factor 16	Fgf16	1.62
prostaglandin reductase 2	Ptgr2	1.59
glycerol-3-phosphate acyltransferase, mitochondrial	Gpam	1.58
spectrin repeat containing, nuclear envelope 1	Syne1	1.56
wingless-type MMTV integration site family, member 4	Wnt4	1.55
Cd44 molecule	Cd44	1.51
tyrosine hydroxylase	Th	1.51
platelet factor 4	Cxcl4/Pf4	1.32
chemokine (C-C motif) ligand 5	Ccl5/Rantes	−1.50
stanniocalcin 1	Stc1	−1.52
antisense paternally expressed gene 3	Apeg3	−1.53
dipeptidylpeptidase 4	Dpp4	−1.64
immunity-related GTPase family, M	Irgm	−1.73
growth hormone releasing hormone receptor	Ghrhr	−1.85
EGF-containing fibulin-like extracellular matrix protein 1	Efemp1	−1.86
ATP-binding cassette, sub-family B (MDR/TAP), member 1A	Abcb1a	−1.89
cartilage acidic protein 1	Crtac1	−2.05
fibroblast growth factor 13	Fgf13	−3.15
SH2 domain protein 1A	Sh2d1a	−4.00
crystallin, gamma B	Crygb	−5.17

Genes were identified by the microarray procedure and their fold-change over control is indicated (gonad from untreated dams) (PF4/Cxcl4 was added in the list as a relevant gene even though its fold change is <1.5).

**Table 3:** Sub-list of characterized genes TCDD-regulated in ovaries.

Gene Title	Gene Symbol	Fold Change
aryl-hydrocarbon receptor repressor	Ahrr	4.20
acyl-CoA thioesterase 1	Acot1	2.94
chemokine (C-C motif) ligand 5	Ccl5	2.50
Zinc finger protein 212	Zfp212	2.42
prolylcarboxypeptidase (angiotensinase C)	Prcp	2.42
leucine rich repeat and Ig domain containing 1	Lingo1	2.22
prostaglandin D2 synthase 2, hematopoietic	Ptgds2/Hpgds	2.00
claudin 15	Cldn15	1.98
leucine rich repeat containing 4C	Lrrc4c	1.95
ras-related C3 botulinum toxin substrate 2	Rac2	1.79
reprimo-like	Rprml	1.78
TBC1 domain family, member 10C	Tbc1d10c	1.76
RT1 class II, locus Ba	RT1-Ba	1.73
histocompatibility 2, class II antigen E alpha	H2-Ea	1.73
granzyme K	Gzmk	1.71
ADAM metallopeptidase domain 10	Adam10	1.70
carbonic anhydrase 5b, mitochondrial	Car5b	1.69
complement component 1, q subcomponent, beta polypeptide	C1qb	1.69
cystatin F (leukocystatin)	Cst7	1.69
chemokine (C-C motif) ligand 6	Ccl6	1.67
C1q and tumor necrosis factor related protein 3	C1qtnf3	1.66
cytochrome P450, family 1, subfamily b, polypeptide 1	Cyp1b1	1.63
HEAT repeat containing 6	Heatr6	1.60
S100 calcium-binding protein A4	S100a4	1.58
platelet factor 4	Pf4/Cxcl4	1.57
potassium inwardly rectifying channel, subfamily J, member 11	Kcnj11	1.52
aldo-keto reductase family 1, member C-like 1	Akr1cl1	1.51
leucine rich repeat containing 61	Lrrc61	−1.51
isoleucyl-tRNA synthetase 2, mitochondrial	Iars2	−1.51
CAP-GLY domain containing linker protein 1	Clip1	−1.51
amphiphysin	Amph	−1.52
ELMO/CED-12 domain containing 2	Elmod2	−1.54
tripartite motif-containing 2	Trim2	−1.54
zinc finger, DHHC-type containing 1	Zdhhc1	−1.54
proline-rich transmembrane protein 1	Prrt1	−1.55
cytoplasmic linker associated protein 1	Clasp1	−1.55
parathyroid hormone receptor 1	Pthr1	−1.55
mutY homolog (E. coli)	Mutyh	−1.56
pinin, desmosome associated protein	Pnn	−1.58
UDP-N-acteylglucosamine pyrophosphorylase 1-like 1	Uap1l1	−1.61
cholecystokinin	Cck	−1.61
sodium channel, voltage-gated, type VII, alpha	Scn7a	−1.61
phosphatidylinositol 4-kinase type 2 beta	Pi4k2b	−1.61
apoptotic chromatin condensation inducer 1	Acin1	−1.62
potassium channel tetramerisation domain containing 13	Kctd13	−1.64
neuronal pentraxin 1	Nptx1	−1.64
mitogen-activated protein kinase 11	Mapk11	−1.68
ATP-binding cassette, sub-family C (CFTR/MRP), member 5	Abcc5	−1.70
purinergic receptor P2X, ligand-gated ion channel, 3	P2rx3	−1.71
S100 calcium binding protein B	S100b	−1.72
ubiquitin-like modifier activating enzyme 7	Uba7	−1.73
Translocase of outer mitochondrial membrane 34	Tomm34	−1.74
ATP-binding cassette, sub-family G (WHITE), member 1	Abcg1	−1.76
family with sequence similarity 92, member A1	Fam92a1	−1.78
solute carrier organic anion transporter family, member 1a4	Slco1a4	−1.81
period homolog 3 (Drosophila)	Per3	−1.83
basic helix-loop-helix family, member e41	Bhlhe41	−1.85
Fc receptor-like S, scavenger receptor	Fcrls	−1.97
phosphodiesterase 10A	Pde10a	−1.98
spastic paraplegia 3A homolog (human)	Spg3a	−2.14
nucleoporin 62 C-terminal like	Nup62cl	−2.47

Genes were identified by the microarray procedure and their fold-change over control is indicated (gonad from untreated dams).

Global gene expression technology provides a comprehensive strategy whereby critical AHR-regulated genes apart from the classic battery of genes can be identified, and has been used to elucidate target pathways involved in the aetiology of TCDD and related compounds toxicity in liver and kidney [15]. Using this strategy, we focused our study on developing gonads, testes and ovaries, in rats. The age of 5 days was chosen for males because this time period coincides with germ cell reentry into mitosis, the set-up of the spermatogonial program including stem cell self-renewal and the maturation of the somatic cell lineages, i.e. the Sertoli and Leydig cells [16]. Regarding females, we concentrated on prepubertal period and specifically infantile period (from 7 to 20 days of age) when the ovary as well as the pituitary displayed an intense endocrine activity with high levels of estradiol and gonadotropins [17], [18]. Real-time PCR was further used not only to validate microarray data generated but also to gain more insight into the kinetics of regulation of the identified genes along with crucial developmental periods. In parallel, the expression level of the classical battery of detoxifying genes was surveyed.

**Table 4:** David functional analysis.

ovarian genes	cluster	enrichment scores	p-value	Examples of genes
up-regulated (34 identified; 4 unknwon)	immune response, positive regulation of celldifferentiation and developmental processes	2.43	4.7.10^−4^	Ccl5, Adam10
	positive regulation of response to stimulus	1.93	5.10^−4^	Ccl5, Ccl6, Adam10
	chemotaxis and chemokine activity	1.7	3.3.10^−4^	Ccl5, Ccl6, Pf4
	response to steroid hormone stimulus and organic substance	1.56	1.2.10^−2^	Adam10, Ccl5, Cyp1b1
down-regulated (56 identified; 19 unknown)	behavior	1.4	2.7.10^−2^	S100a4, Amph, Cck

Summary of the analysis using the list of genes regulated by TCDD in ovary.

Overall, our results evidenced that both sex gonads responded differently to TCDD exposure with respect to enzymes of the detoxifying machinery. We illustrated that inflammatory pathway is one pathway activated by TCDD in gonads. We identified several new genes targeted by TCDD including Fgf13 in testis and one gene, Ptgds2/Hpgds regulated in the two sex gonads.

**Table 5:** Expression levels of classical genes targeted by TCDD.

	ovary			Testis		
	control	TCDD	Fold-Change	control	TCDD	Fold-change
Ahrr	18	79	4.2*	14	48	3.34*
Cyp1a1	nd	nd	nd	nd	nd	nd
Cyp1a2	nd	nd	nd	nd	nd	nd
Cyp1b1	1419	2300	1.63*	1079	1315	1.22
Cyp2s1	66	55	0.83	65	54	0.83
Nqo1	350	470	1.34*	303	444	1.46
Gsta1	13200	12800	0.96	7705	7652	0.99
Aldh3a1	nd	nd	nd	nd	nd	nd
Ugt1a6	210	273	1.3	381	436	1.14

Data were obtained using ovaries of 14 days of age and testes of 5 days of age.

* *p*<0.05; nd, not detectable.

## Materials and Methods

### Experimental Design

Time pregnant Sprague-Dawley females of embryonic day 12 were purchased from Janvier’s Breeding (Le Genest, France). They were housed individually in plastic cages with food (Altromin 1310; Genestil, Royaucourt, France) and water provided ad libitum at 23°C and a 12∶12 photoperiod. Animals were randomly assigned to treatment groups. Dams were allowed 3-day acclimatization and were given one oral dose of 2,3,7,8-TCDD (ref ED-901-C) (LGC Promochem, Molsheim, France) 200 ng/kg body weight (bw) in sesame oil on embryonic day 15. Control animals received sesame oil. Pups were sacrificed by cervical dislocation under CO_2_ anesthesia at various ages, at 3, 6, 10, 12, 14 and 25 days for females, and at 5, 28, 40, 67, 145 days for males. Ovaries and testes were dissected and snap-frozen. Pituitaries were collected from female pups of 3, 6, 12 and 14 days of age. In addition, livers were collected at 5 and 28 days for males and 3 and 6 days for females. All organs were kept at −80°C before use. Throughout the manuscript, treated organs refer to organs collected from animals born from TCDD-exposed dams. Control organs refer to organs collected from animals born from sesame oil exposed dams. Animals were housed and maintained according to published European communities’ guidelines (86/609/CEE) and all the performed experiments on animals were approved by the experimental animal committee of the Paris Rive Gauche site (Agreement A75-13-17, Centre National de la Recherche Scientifique, Paris 7 University, Paris, France). A detailed protocol of TCDD exposure and follow-up of the animals has been published [19].

**Figure 2:**
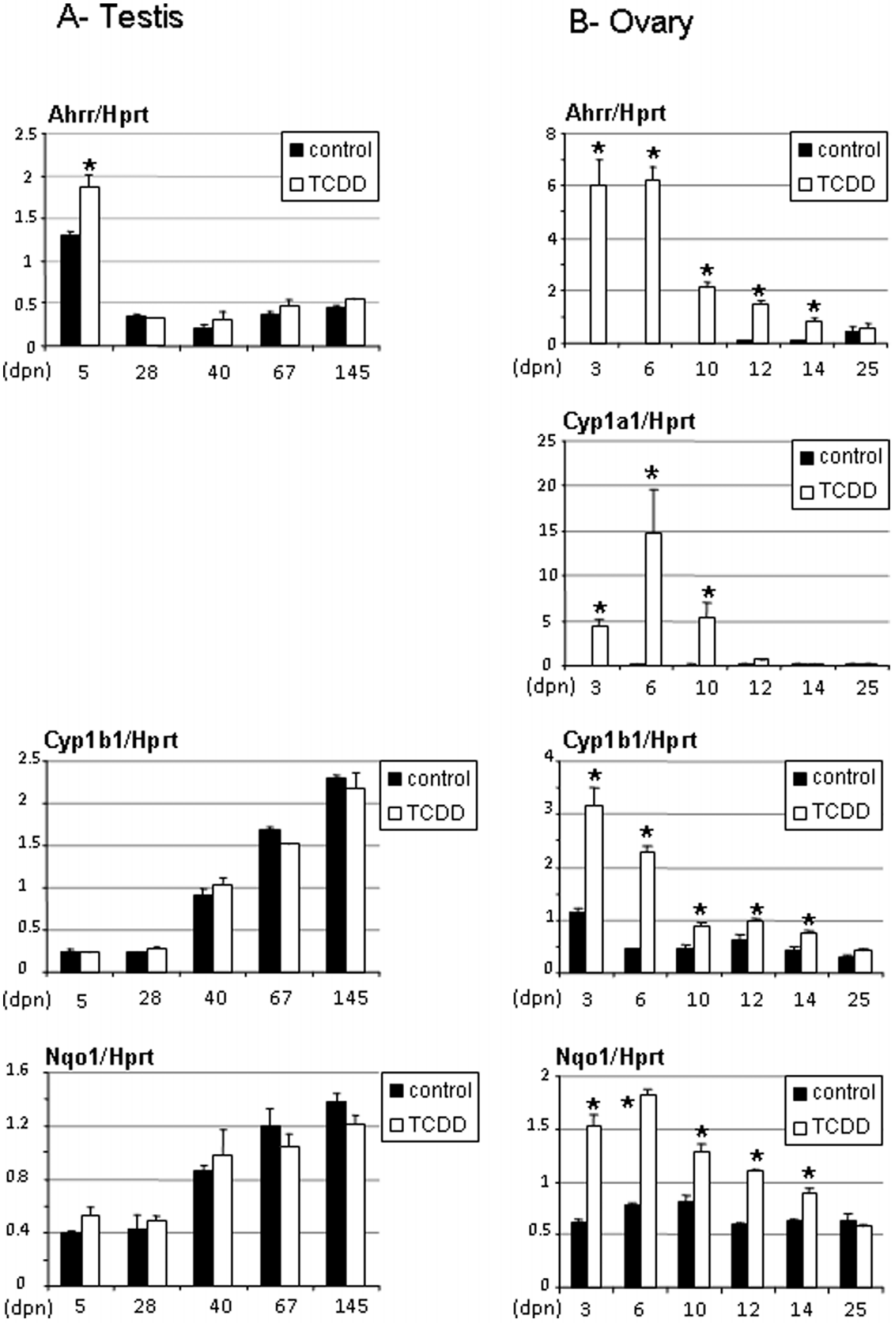
Ahrr, Cyp1a1, Cyp1b1 and Nqo1 gene expression levels in testes and ovaries of rats exposed in utero to TCDD. Testes were recovered from males aged from 5 to 145 days (A) and ovaries were recovered from females aged from 3 to 25 days (B). Testes samples did not have detectable Cyp1a1 levels (A). Levels were normalized using Hprt. Values are the mean ± SEM of n = 4 to 6 animals (n = 2 for 3- and 25-day control ovaries). *p<0.05 versus its time-matched control. (dpn), days postnatal.

### RNA Preparation and Microarray Analysis

Changes in global gene expression induced by TCDD were analyzed in 5-day old testes and in 14-day old ovaries. In both cases, 3 rats treated in utero by TCDD were compared to 3 rats treated with sesame-oil vehicle. RNA extraction was performed with the RNeasy Mini RNA extraction kit (Qiagen, Courtaboeuf France).

**Figure 3:**
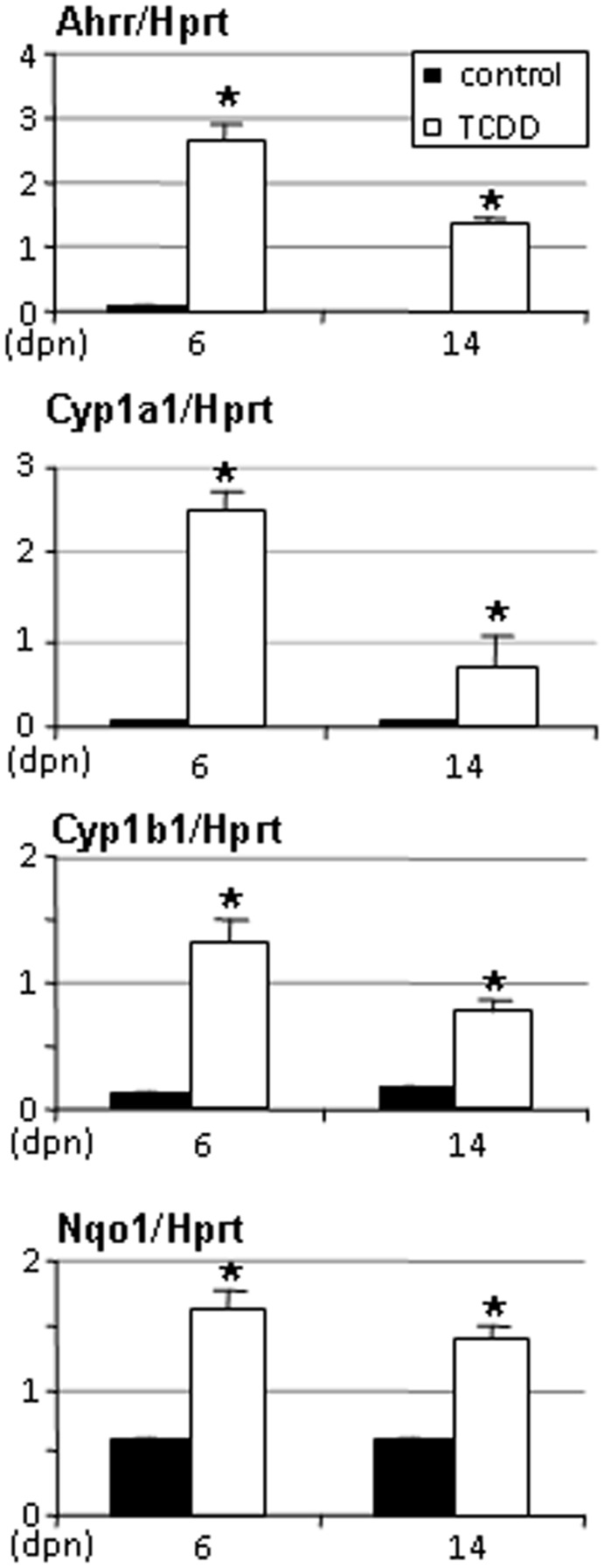
Ahrr, Cyp1a1, Cyp1b1 and Nqo1 gene expression levels in pituitaries of rats exposed in utero to TCDD. Pituitaries were recovered from females aged from 6 and 14 days of age. Levels were normalized using Hprt. Values are the mean ± SEM of n = 3 to 5 animals. * p<0.05 versus its time-matched control. (dpn), days postnatal.

Microarray analysis has been performed in the genomic and microgenomic core facility profileXpert (Bron, France) as described previously [20], [21], using a high-density oligonucleotide array (GeneChip Rat Genome 230 2.0 array, Affymetrix, Santa Clara, CA, USA), and following Affymetrix protocol (http://www.affymetrix.com). The arrays were read with a confocal laser (Genechip scanner 3000, Affymetrix). Then CEL files were generated using the Affymetrix GeneChip Command Console (AGCC) software 3.0. The obtained data were normalized with Affymetrix Expression Console software using MAS5 statistical algorithm. Normalized data were compared and filtered using Partek Genomic Suite software 6.5 (Partek Inc., St. Louis, MO, US). Microarray data are available in the GEO database under the number GSE32890.

**Figure 4:**
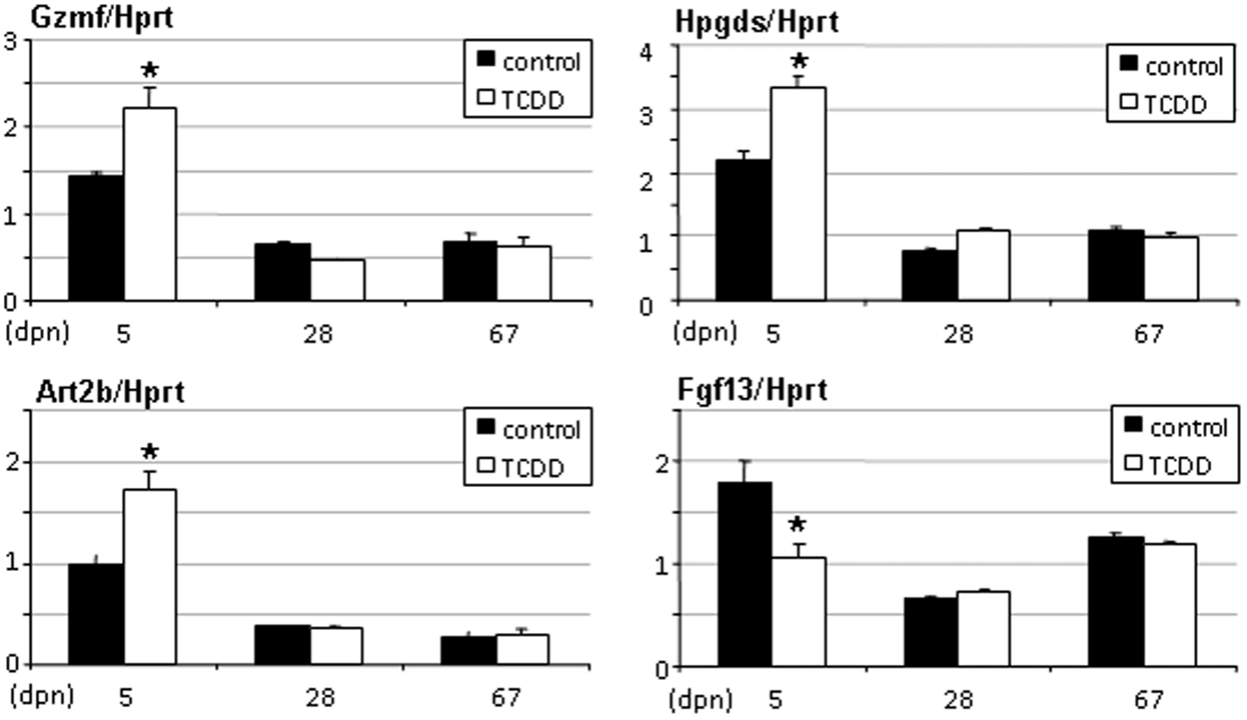
Gzmf, Art2b, Hpgds and Fgf13 gene expression levels in testes of rats exposed in utero to TCDD. Testes were recovered from males of 5, 28 and 67 days of age. Levels were normalized using Hprt. Values are the mean ± SEM of n = 4 animals. *p<0.05 versus its time-matched control. (dpn), days postnatal.

### Analysis of microarray data

A principal component analysis (PCA) of the complete list of genes present in the chip and two sample t-test were performed between TCDD samples and control samples for ovaries and for testes. Only genes showing a variation of at least 1.5-fold and a p-value less than 0.05 were retained. Then, a gene was considered as differentially expressed between groups only if the detected signal was above the background for at least one of the compared groups. David functional annotation clustering (http://david.abcc.ncifcrf.gov/conversion.jsp) was performed to identify enrichment in biological functions and pathways. Promoter sequences (650pb) of the regulated genes were extracted from Rat RGSC 3.4 assembly using BioMart, and firstly analyzed for pattern matching using Common TFs from the Genomatix software package (Genomatix software suite v2.5, München, Germany). Briefly, the sequences were scanned for matches to Transcription Factor binding sites and only matrices displaying an enrichment p-value <0.05 were considered enriched in the promoters of interest compared to the rest of the vertebrate promoter sequences.

**Figure 5:**
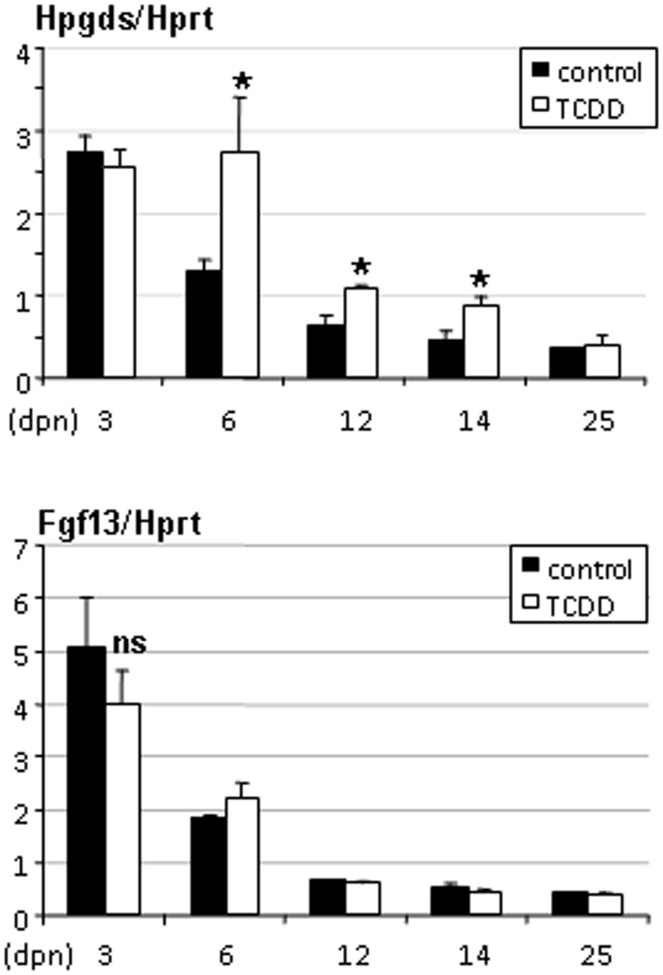
Hpgds and Fgf13 gene expression levels in ovaries of rats exposed in utero to TCDD. Ovaries were recovered from females of 3 to 25 days of age. Levels were normalized using Hprt. Values are the mean ± SEM of of n = 4 to 6 animals (n = 2 for 3- and 25-day control ovaries). *p<0.05 versus its time-matched control. (dpn), days postnatal.

### Reverse Transcription and Real-time RT-PCR

Reverse transcription was carried out with 250 ng of total RNA recovered from testes (of 5, 28, 40, 67, 145 days of age), ovaries (of 3, 6, 10, 12, 14, 25 days of age), pituitaries (of females aged of 3, 6, 12, 14 days of age) or livers (of 5 and 28 days for males and 3 and 6 days for females), 400 ng oligo-dT primers (Qiagen) and Superscript II reverse transcriptase (Invitrogen, France). Real-time RT-PCR (QRT-PCR) was performed in a LightCycler 480 instrument (Roche Diagnostics, Mannheim, Germany) using the LightCycler 480 SYBR Green I Master mix according to the manufacturer’s protocol. Primers used are listed in table 1. They were tested before use for specificity and efficacy. Amplification conditions were as following: 10 min at 95°C followed by 40 cycles of denaturation (10 sec at 95°C), annealing (10 sec at 60°C), and extension (10 sec at 72°C) with single acquisition of fluorescence at the end of each extension step. The specificity of PCR products was confirmed by analysis of the melting curve and agarose gel electrophoresis. All samples were run in quadriplicate reactions (duplicate of two dilutions). Quantification of gene expression was performed using the Relative Quantification Software (Roche Diagnostics), and data were expressed as a ratio of target gene to the reference gene hypoxanthine phosphoribosyltransferase, HPRT. Statistical analyses were done using Statview 5.0 software package (SAS Institute Inc. Cary, NC 27513). Comparisons between treatments were made by one-way analysis of variance (ANOVA) followed by the post hoc Fisher PLSD test for multiple comparisons. A *p* value of less than 0.05 was considered significant.

**Figure 6:**
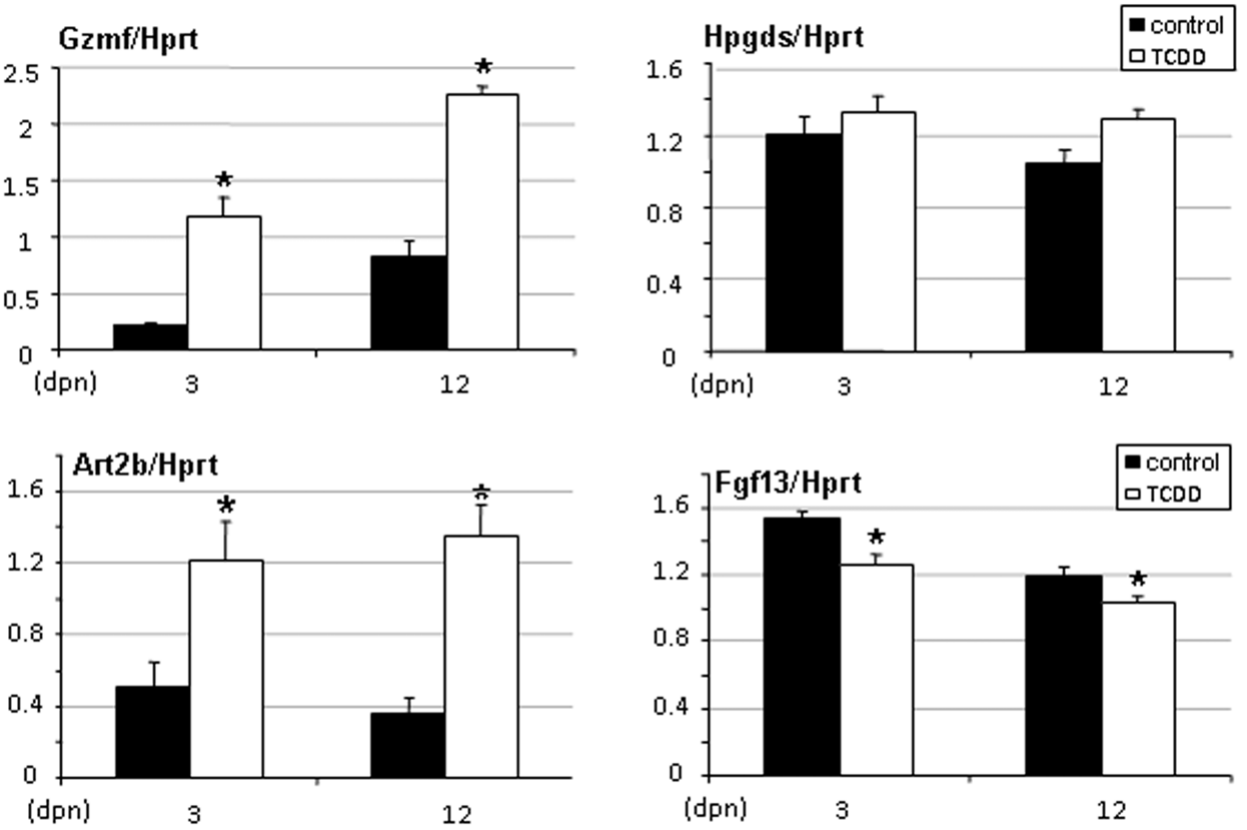
Gzmf, Art2b, Hpgds and Fgf13 gene expression levels in pituitaries of rats exposed in utero to TCDD. Pituitaries were recovered from females of 3 to 12 days of age. Levels were normalized using Hprt. Values are the mean ± SEM of n = 4 animals. *p<0.05 versus its time-matched control. (dpn), days postnatal.

**Figure 7:**
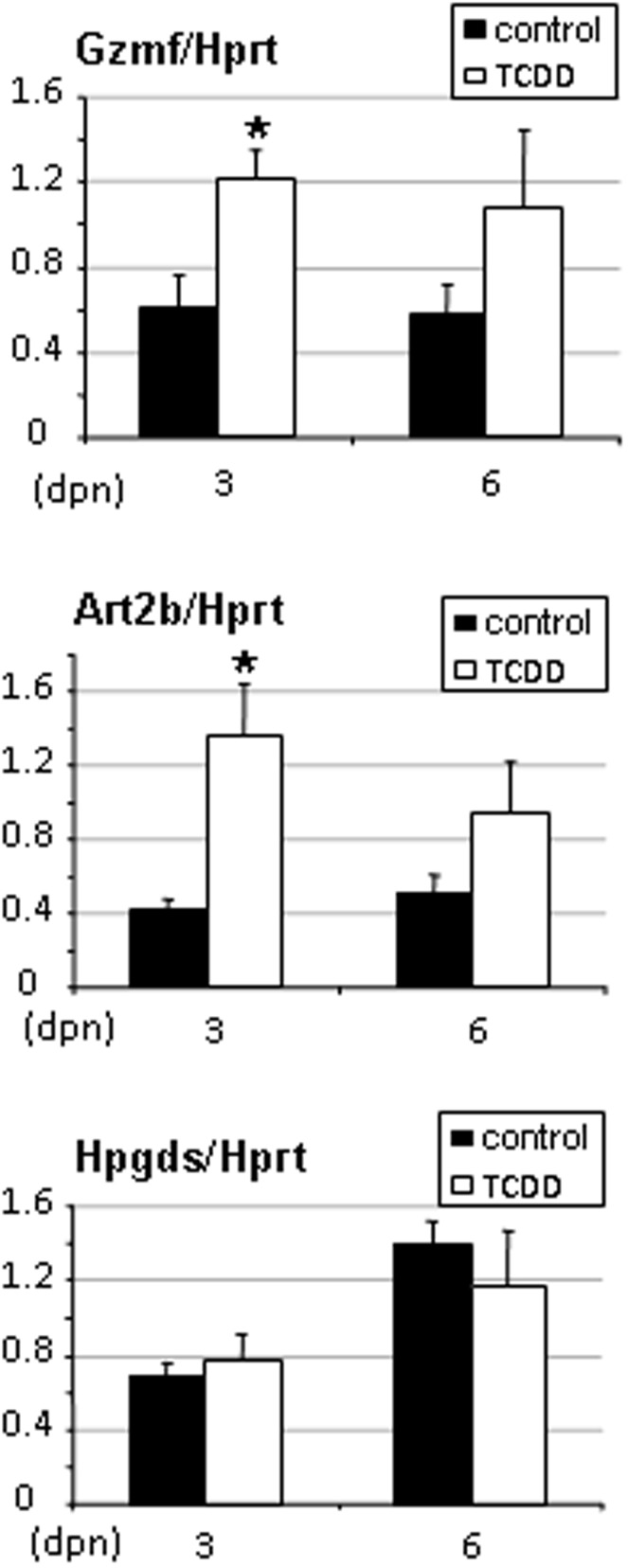
Gzmf, Art2b, and Hpgds gene expression levels in livers of rats exposed in utero to TCDD. Livers were recovered from females of 3 and 6 days of age. Levels were normalized using Hprt. Values are the mean ± SEM of n  = 3 to 5 animals. *p<0.05 versus its time-matched control. (dpn), days postnatal.

**Table 6:** Summary of the effects on Art2b, Fgf13, Gzmf and Ptgds2/Hpgds following TCDD exposure.

	Testis	Ovary	Pituitary	Liver
**Ptgds2/** **Hpgds**	up-regulated at 5 but notat 28 and 67 days of age	up-regulated at 6, 12 and14 but not at 3 or 25 daysof age	expressed but not regulatedat 3 and 12 days of age	expressed but not regulated at 3 and 6 days of age
**Fgf13**	down-regulated at 5 but notat 28 and 67 days of age	expressed but not regulatedfrom 3 to 25 days of age)	down-regulated at 3 and12 days of age	not detected at 3 and 6 days of age
**Gzmf**	up-regulated at 5 but notat 28 and 67 days of age	not detected	up-regulated at 3 and 12 daysof age	up-regulated at 3 but not at 6 days of age
**Art2b**	up-regulated at 5 but notat 28 and 67 days of age	not detected	up-regulated at 3 and 12 daysof age	up-regulated at 3 but not at 6 days of age

## Results

### Reproductive Parameters of the F1 Offspring

A follow-up of the female offspring including body weight, fertility assessment and measurement of mRNA levels of some key genes involved in the endocrine function of the ovary during prepubertal period is provided (Supporting Information S1). Except for body weight, no significant differences were obtained. Regarding the male offspring exposed in utero to TCDD, testicular and epididymal weight did not change. Testis histology was grossly normal so were testosterone levels throughout development. A precise description was provided previously [19].

**Table 7:** List of Matrices Family overrepresented in the promoter sequences of the TCDD regulated genes in testes.

DNA Matrices Family	Transcription factors	p-value	#sequences
**V$ETSF**	Ehf, Elf1,Elf2, Elf3,Elf4, Elf5,Elk1, Elk3,Elk4,Erf, Erg, Ets1, Ets2, Etv1,Etv2, Etv3,Etv4,Etv5,Etv6, Fev,Fli1, Gabpa,Gabpb1, Gabpb1l,Gabpb2, Sfpi1, Spdef, Spib,Spic	0.0127746	25
**V$GCMF**	Gcm1, Gcm2	0.0072138	22
**V$MYT1**	Myt1, Myt1l,St18	0.0355996	22
**V$CDXF**	Cdx1, Cdx2,Cdx4	0.00668855	21
**V$SF1F**	Nr5a1, Nr5a2	0.00610062	16
**V$RREB**	Rreb1	0.0090441	15
**V$GRHL**	Grhl1, Grhl3	0.0176176	14
**V$INSM**	Insm1	0.0473584	13
**V$LTFM**	Ltf	0.00939393	12
**V$NBRE**	Nr4a1, Nr4a2,Nr4a3	0.0365125	10
**V$PAX9**	Pax9	0.0173214	8

**Table 8:** List of Matrices Family overrepresented in the promoter sequences of the TCDD regulated genes in ovaries.

DNA Matrices Family	Transcription factors	p-value	#sequences
**V$ETSF**	Ehf, Elf1,Elf2, Elf3,Elf4, Elf5,Elk1, Elk3,Elk4, Erf,Erg Ets1, Ets2, Etv1,Etv2, Etv3,Etv4, Etv5,Etv6, Fev,Fli1, Gabpa,Gabpb1, Gabpb1l, Gabpb2, Sfpi1,Spdef,Spib,Spic	0.00212351	54
**V$SP1F**	Cdca7l, Eapp,Klf10, Klf11,Klf5, Sp1,Sp2, Sp3,Sp4, Sp5,Sp6, Sp7,Sp8	0.000099594	46
**V$MYBL**	Myb, Mybl1,Mybl2	0.044378	46
**V$IRFF**	Irf1, Irf2,Irf3, Irf4,Irf5, Irf6,Irf7, Irf8,Irf9, Rnf31	0.0422676	44
**V$ZF02**	Zbtb7a, Zbtb7b,Zfp148, Zfp202, Zfp219,Zfp281	0.0076816	41
**V$EGRF**	Egr1, Egr2,Egr3, Egr4,Wt1	0.0218414	38
**V$MAZF**	Maz, Zfp278	0.013593	33
**V$CTCF**	Ctcf, Ctcfl,LOC100360757	0.0329843	33
**V$PAX2**	Pax2	0.0270729	27
**V$ZF10**		0.0137818	23
**V$BTBF**	Zbtb33	0.0416754	14

### Global Analysis of Testes and Ovaries Transcriptomic Data

The total amount of genes expressed in ovaries and testes was approximately of 65% of the rat genome, indicating that an average of 20,000 genes is expressed in the gonads. PCA analysis did not allow segregating genes between treated-testes and control testes, or between treated-ovaries and control ovaries. It indicated that the sample to sample variation within the group (male or female) was bigger than the variation due to the treatment (not shown). A two sample t-test was next performed and a list of 113 and 56 differentially expressed genes showing a variation of 1.5-fold and a p-value less than 0.05 came out in ovary and testis, respectively. Of the 113 genes responding to TCDD in ovaries, 38 were up-regulated and 75 down-regulated. In testes, 27 were up-regulated and 29 down-regulated (Fig. 1). About, half of them are not fully characterized and are identified by their Affymetrix probeset ID. Some of them are transcribed locus or expressed sequence tags (ESTs). A sub-list of characterized genes differentially expressed in response to TCDD, in testes and in ovaries is given in Tables 2 and 3, respectively. We also identified a total of 7 genes common to testes and ovaries if cross-comparing the list of regulated genes that exhibited at least a 1.5 fold change over its respective control. There were Ahr repressor (Ahrr), prostaglandin D2 synthase 2, hematopoietic (Ptgds2/Hpgds), the chemokines Rantes/Ccl5 and Pf4/Cxcl4, and 3 uncharacterized genes which were not further studied (Fig.1, Tables 2, 3).

David functional annotation clustering indicated one cluster with enrichment score of 1.61 and a p-value of 4.9.10^−3^ using the total list of testis genes. It corresponded to immune and defense response with 6 transcripts including Ccl5 and Pf4. No cluster came out if only the list of the up or down-regulated genes was considered. Using the list of the up-regulated genes in the ovary, 4 clusters came out with enrichment score comprised between 1.56 and 2.43 and corresponding to immune response, positive regulation of response to stimulus, chemotaxis and chemokine activity, and response to steroid hormone stimulus. In addition, KEGG pathway pointed to the chemokine signaling pathway associating Ccl5, Ccl6 and Pf4. A single cluster with an enrichment score of 1.4 and corresponding to behavior came out when uploading the list of down-regulated genes (Table 4).

### Expression of Genes Belonging to the Classic Battery of TCDD Target Genes

To extend microarray data, we first focused on Ahrr strongly induced in both gonads, and other genes belonging to the TCDD-inducible Ahr gene battery. The TCDD battery includes in addition to Ahrr which acts as a dominant negative factor to repress Ahr induced signalling pathways [22], [23], four phase I xenobiotic metabolizing enzymes, i.e., Cyp1b1, Cyp1a1, Cyp1a2, and Cyp2s1, and four phase II xenobiotic metabolizing enzymes, i.e., NADP(H) quinone oxidoreductase (Nqo1), glutathione transferase a1 (Gsta1), cytosolic aldehyde dehydrogenase-3 (Aldh-3a1), and UDP glucuronosyltransferase 1a6 (Ugt1a6) (Table 5). Three genes, Cyp1a1, Cyp1a2 and Aldh-3a1 were not found at detectable levels in the gonads. Two genes appeared to be constitutively expressed (Cyp2s1 and Gsta1) or weakly enhanced (Nqo1 and Ugt1a6) after TCDD treatment. Cyp1b1 was significantly up-regulated in ovaries (FC 1.63) but not in testes (FC 1.22 with a *p*-value of 0.36). Finally, gene coding Ahrr was the most up-regulated gene in both gonads of treated animals (FC 4.2 in ovaries and 3.34 in testes).

To validate and extend the microarray results, we analyzed the expression of Ahrr and Cyp1b1 as well as Cyp1a1 and Nqo1 using real-time PCR. This was done not only in ovaries at 14 days and in testes at 5 days but also during a period of time extending from 3–25 days in females and 5–145 days in males to cover critical developmental steps in both gonads. Real-time PCR confirmed the findings of the microarray in gonads (Fig. 2). For example in testes, Ahrr was significantly enhanced at 5 days of age although the fold-change was lower than the one observed using microarray. In addition, none of the metabolizing enzymes had their gene expression levels altered after TCDD treatment (Fig. 2). Noticeably, Cyp1a1 gene was not expressed in testis, either control or TCDD-treated (not shown). Cyp1b1 and Nqo1 expression levels increased as a function of age from 5 to 145 days (Fig. 2). In contrast, neonate ovaries recovered from the same litters were strongly responsive to TCDD exposure. Expression levels of Ahrr were maximally up-regulated at 3 and 6 days. A significant enhancement was still observed until 14 days, consistent with the microarray data. No effect was observed in ovaries of 25 days. Of note, Ahrr gene expression levels were almost undetectable in control ovaries (Fig. 2). Cyp1a1, the hallmark of TCDD-inducible Ahr gene [24], although not present in control ovaries was highly induced in TCDD-treated ovaries of 3 to 10 days, peaking at 10 days. No expression was detected at 14 days consistent with the microarray data (Table 2). Gene expression levels of Cyp1b1 and Nqo1 were at detectable levels in control ovaries and roughly constant from 3 to 25 days of age. Treated ovaries had enhanced levels of Cyp1b1 and Nqo1 from 3 to 14 days, also in agreement with the microarray data. No differences were observed at 25 days (Fig. 2).

We also studied an additional endocrine organ, the pituitary, and the liver as the primary detoxifying organ. Data synthesized on Fig. 3, indicate a strong and significant enhancement of Ahrr, Cyp1a1, Cyp1b1 and Nqo1 at 6 days of age in pituitary of TCDD-treated animals. Although significant, induction was less pronounced at 14 days of age (Fig. 3). As expected, RNA from both male (5 days old) and female (6 days of age) liver showed increased Ahrr and Nqo1 expression levels (data not shown) in treated animals consistent with the induction in Cyp1a1 mRNA levels reported previously [19].

### Expression of Chemokines in Response to TCDD in Gonads but also in Pituitary and Liver

Consistently with our previous data [19], we observed down-regulation of Ccl5 and upregulation of Pf4/Cxcl4 in the testes of 5-day old rats of TCDD-treated dams (Table 2). In females, microarray studies indicated that both Ccl5 and Pf4/Cxcl4 were significantly up regulated 2.5- and 1.57-fold, respectively (Table 3). In addition, KEGG pathway pointed to the chemokine signaling pathway associating Ccl5, Ccl6 and Pf4/Cxcl4, in ovary and one enriched cluster comprised of Ccl5 and Pf4/Cxcl4 in testis. To define if chemokines could be targeted by TCDD in other organs, real-time PCR was performed with RNA from pituitary and liver. We found that pituitaries recovered from 14-day-old female rats from TCDD-treated dams had increased Ccl5 (3.26 fold-change, n = 4; p<0.05) and Pf4/Cxcl4 (1.81 fold-change, n = 4; p<0.05) versus time-matched controls. Ccl5 but not Pf4/Cxcl4 was also up-regulated (2.12 fold-change, n = 4; p<0.05) in the liver recovered from 28-day old male rats (data not shown).

### TCDD-regulated Genes in Testis and Comparison with Two Endocrine Organs (Ovary and Pituitary) and Liver

Microarray data were further exploited by real-time PCR to analyse the testis response to TCDD exposure. Genes selected included Art2b, Gzmf/Nrkp7, and Fgf13 because they were among the most regulated genes in testis, and Ptgds2/Hpgds because it was regulated in both sex gonads (Table 2). We observed up-regulation of Gzmf/Nrkp7, Art2b, Ptgds2/Hpgds and down-regulation of Fgf13 (p<0.05) at 5 but not at 28 or 67 days (Fig. 4).

In ovaries, Ptgds2/Hpgds was present at all ages investigated from 3 to 25 days. Enhanced expression levels of Ptgds2/Hpgds were observed in the ovaries of rats from TCDD-treated dams at 6, 12 and 14 days but not at 3 and 25 days of age (Fig. 5). Fgf13 was present in neonate ovaries (3 and 6 days of age) but its expression levels did not change in treated ovaries (Fig. 5). Art2b and Gzmf/Nrkp7 were neither expressed in the ovary at 6, 10 and 14 days of age, nor induced in the ovaries of rats from TCDD-treated dams at these age-time points (not shown).

In pituitaries, Gzmf/Nrkp7 and Art2b were present at 3 and 12 days of age and organs from treated animals exhibited levels higher than 2-fold over control levels (Fig. 6). Fgf13 was also present in pituitaries collected from 3 and 12 days old animals, and its expression levels were significantly decreased (a 20% decrease) in treated organs (Fig. 6). Ptgds2/Hpgds was expressed in pituitary but not TCDD-regulated (Fig. 6). Finally, real-time PCR using liver from 3 and 6 day-old animals revealed transcripts for Gzmf/Nrkp7, Art2b and Ptgds2/Hpgds but not Fgf13, with TCDD up-regulation of Gzmf/Nrkp7 and Art2b (p<0.05) at 3 but not at 6 days of age. Ptgds2/Hpgds was not targeted (Fig. 7). Table 6 recapitulates data.

### Identification of DNA Matrices, Potential Sites for Transcription Factors

To find out whether certain transcriptional factor binding site(s) were enriched in the TCDD-regulated genes identified in this study, we selected 650bp promoter of each gene, and searched for DNA matrices families using Genomatix promoter analysis. A total of 25 promoter sequences for testes regulated genes and 54 promoter sequences for ovaries regulated genes could be processed. We observed that the DNA matrices family ETSF was consistently identified in the promoters of all 25 TCDD regulated genes in testes (Table 7) and the 54 regulated in ovaries (Table 8). We also identified 10 other overrepresented matrices families’ specific to either testes or ovaries (Table 8) which may account for the gender differences that we detected in this study.

## Discussion

The objective of our project was to determine the impact of an in utero exposure to 2,3,7,8-tetrachlorodibenzo-p-dioxin (TCDD) on reproductive function of male and female offspring in the rat with a special emphasis on the immature period, in conditions in which reproductive parameters are grossly normal both in males [19] and in females (this study). To this end, animals were exposed during gestation to low doses to avoid collateral toxic effects. We also considered the TCDD half-life of 3 weeks in rodents, and conclusions published [25] indicating that the majority of occurrences of TCDD in offspring of dosed dams arise from lactational transfer of TCDD. In addition, given the unexpected microarray results highlighting gender difference responses; we developed a real time PCR approach on gonads at various developmental periods of interest. On the one hand, this approach permitted to extend our study on male and female gonad development following an in utero exposure to TCDD. On the other hand, this approach allowed comparing common stages in both sexes (specifically, 5 and 28 days in testes, and 6 and 25 days in ovaries).

The microarray technology is a powerful method allowing full gene analyses of different samples. It is especially fruitful when scarce differences are expected. In the present study, less than 1% of the expressed genes in gonads were found to be altered following embryonic TCDD exposure. Specifically, we identified a total of 113 genes in ovaries and 56 genes in testes differentially regulated by the treatment over the 20,000 genes found expressed in the ovary and testis, respectively. The low number of regulated genes found in testis versus the ovary probably suggested that TCDD may have less deleterious effects in testes than in ovaries. This could result from the lack of response of the TCDD battery of detoxifying genes in testis by comparison with the neonate ovary. Indeed, TCDD only induced gene expression of Ahrr in neonate testes while in ovaries, Cyp1a1, Cyp1b1, and Nqo1 exhibited a very high up-regulation in addition to Ahrr. Ahrr functions as a naturally occurring dominant-negative factor [22], [23]. Hence, Ahrr is an important determinant of tissue specific responsiveness to TCDD, and strong evidences have been reported on an inverse relationship between Ahrr expression and sensitivity to induction of xenobiotic-metabolizing enzymes caused by TCDD. This is coherent with elevated constitutive levels of Ahrr in testis [26], [27]. The data presented herein i.e., high expression of Ahrr and no detectable Cyp1a1, Cyp1b1 and Nqo1 induction in testis are in keeping with these observations.

Nonetheless, the presence of Ahrr and its up-regulation in TCDD treated testes indicate that the Ahr canonical pathway is active in testis even though the role played by Ahr in testis is misunderstood. Impairment of the urogenital sinus is the major phenotype in Ahr^−/−^ male mice [8], [28]. Testis alteration was also reported in aged Ahr^−/−^ mice with reduced testosterone production and sperm numbers [29]. It is of interest that enzymes of the TCDD battery of inducible genes expressed in testis including Cyp1b1 and Nqo1 had their expression levels increasing as a function of time, from infancy to adulthood. It may suggest that these enzymes, which are confined to Leydig cells in testis [30], [31], exert a physiological role during development in relation with the endocrine status of the animal.

Our data also illustrated that in addition to liver, which is a primary detoxification organ, endocrine organs such as ovary and pituitary displayed an up-regulation of the expression of genes coding enzymes of the detoxifying machinery. For example, in both ovary and pituitary there was an induction of Cyp1a1 which is the hallmark of TCDD exposure [24]. Nonetheless, Cyp1a1 induction was no longer observed at 12 days of age in the ovary while still detected at 14 days in pituitaries (and 25 days, not shown) and 28 days in livers [19], illustrating organ specificity in the timing of the response. Indeed, the other detoxifying genes studied in ovary, i.e., Ahrr, Cyp1b1 and Nqo1 were still enhanced at 12 and 14 days of age. Together, these data are in keeping with previous studies demonstrating enhanced Ahrr and Cyp1a1 in the pituitaries of male rats exposed to an acute-dose of TCDD at the adult age [32]. Regarding the ovary, evidences have been brought indicating enhanced Cyp1a1 in response to dioxin [33]. However, to our knowledge, this is the first report illustrating the TCDD-induction of Ahrr in ovary.

Present data extend our previous study reporting alteration of chemokines in the testes of TCDD-treated rats, with up-regulation of Cxcl4 and down-regulation of Ccl5 [19], and other studies pointing out that various chemokines were targeted by TCDD exposure including Ccl5 in a model of endometriosis [34], Ccl1 [35], Ccl2 [36]. In this study, the chemokine pathway associating Ccl5, Pf4/Cxcl4 and Ccl6 was induced in TCDD-treated ovaries. Therefore, in addition to growth factors and cytokines [37], chemokines might be added to the list of the TCDD-targeted genes, extending the notion that TCDD interacts with the inflammatory pathways.

Our results also evidenced a sex specific response of gonads to the TCDD exposure with Fgf13, Art2b, and Gzmf regulated in testes but not in ovaries, and, a gonad gene expression signature with Ptgds2/Hpgds. Indeed, this study demonstrated that Ptgds2/Hpgds, although expressed in various tissues, was only regulated by TCDD in the gonads. Interestingly, none of these genes, and Ccl5 and Pf4/Cxcl4 exhibited-XRE elements in the 2,0000 bp upstream of the transcription sites, in rats (not shown). This situation contrasted with the mouse orthologs of Art2b, Fgf13, Ptgds2/Hpgds and Ccl5 having XRE elements in the 2,000 bp upstream of the transcription sites (http://drgap.nies.go.jp/pub/page/element). Nonetheless, these genes may be direct targets if considering that only 1/3 of the gene expression responses elicited by TCDD involves direct AhR binding to a XRE [11]. It may also indicate that the TCDD-induced enhancement of these genes is secondary to a primary event, which remains to be defined. It may well be the case for Ptgds2/Hpgds because its induction in ovary was not immediate and could not be detected at 3 days of age, an age at which all the detoxifying genes have been shown to be deregulated. We cannot ascertain that this situation is female-specific because we did not recover males younger than 5 days of age.

Ptgds2/Hpgds is a cytosolic protein responsible for the biosynthesis of prostaglandin D2 (PGD2) in immune and inflammatory cells, being widely distributed in antigen presenting cells [38]. Interestingly, very recent data have brought new insight into involvement of Ptgds2/Hpgds in differentiation and function of gonads [39], [40]. Ptgds2/Hpgds is expressed in the early embryonic gonad in both sexes and participates to the initial nuclear translocation of the Sox9 protein triggering Sertoli cell differentiation in males [40]. It is also expressed in the adult ovary where it indirectly participates to the regulation of progesterone secretion [39]. Therefore, more studies need to be done regarding to Ptgds2/Hpgds because of the stimulatory effect of PGD2 on steroidogenesis regulation [39].

The 3 other genes, Fgf13, and Gzmf and Art2b, were among the most differentially expressed genes in testis, in response to TCDD. Fgf13 (also called Fgf homologous factor 2) was first shown to be expressed in human fetal and adult brain and in adult kidney [41]. Fibroblast Growth Factors (FGFs) form a large family of conserved signaling proteins with essential developmental functions in organ patterning and morphogenesis. Fgf13 belongs to the intracrine FGF family indicating that it is not secreted and that it functions in an FGF receptor-independent manner [42]. In fetal mice, Fgf13 expression was detected within the mesonephros of both sexes at 12.5 embryonic day, and restricted to testis by embryonic day 13.5 [43], [44]. In the present study, Fgf13 showed high inhibition in testis microarray and data were validated by real time PCR with gene expression levels halved in testis from TCDD-treated rats. Interestingly, its expression was not regulated in ovaries while down- regulated in pituitary; it may suggest that Fgf13 is under the regulation of transcription factors commonly expressed in testis and pituitary.

There is little information regarding Gzmf and Art2b and their possible regulation by TCDD. Granzyme genes are serine proteinases previously shown to be implicated in tissue remodelling in the placenta and in the testis [45], [46]. Art2b is an ADP-ribosyl transferase gene, and 5 Arts have been described in the mammalian genome. ADP ribosylation is a reversible post-translational modification that can be used as a mechanism to regulate endogenous functions [47]. The full significance of the alteration of these genes in the testis and pituitary, but not in the ovary recovered from siblings’ warrants further investigation. Interestingly, through cross-comparison between the lists of DNA matrices present in the proximal promoters of 25 genes in testes and 54 genes in ovaries, all regulated by TCDD, we extracted potential families of transcription factors allowing regulation by TCDD in testis and/or ovary. However, the importance of these findings remains to be determined.

Overall, our results evidenced that male and female gonads responded differently to TCDD exposure showing, for example, the induction of the canonic battery of genes coding enzymes of the detoxifying machinery in ovaries but not in testes. We illustrated that inflammatory pathway is one of the pathways targeted by TCDD in gonads. Finally, we identified several new genes targeted by TCDD, including Fgf13 in testis and one gene, Ptgds2/Hpgds targeted in testis and ovary.

## Supporting Information

Supporting Information S1 **Reproductive parameters of the female progeny exposed in utero to TCDD. (a) F1 female progeny weight from 5 to14 postnatal days.** Values (g) are mean ± SEM of () number of pups. TCDD-200 ng females were significantly lighter than control females at 4, 7 and 10 postnatal days (* *p*<0.05). **(b) F1 female fertility assessment.** Control females (7) and TCDD-200 ng females (5) were mated continuously with males from 2 to 7 months of age. A total of 5 litters was obtained for each female. Newborn pups were sacrificed after 2 days to check viability. For each litter, the mean number of days between beginning of mating and parturition is indicated for the 7 control and 5 TCDD-treated females. We also recorded the mean number of pups of each sex for the 7 control and 5 TCDD-treated females. **(c) Expression levels of key genes involved in endocrine function of the ovary.** No significant differences were observed between control and TCDD-200 ng treated females assessed through a transcriptomic analysis on 14 dpn ovaries. Values are the mean of the 3 samples analysed by microarray. **(d) Real-time RT-PCR measurement of Cyp19a1 and Star genes in ovaries during prepubertal period.** Values were normalized using Hprt and are mean ± SEM of () number of ovaries. No significant differences were observed between control and TCDD- 200 ng treated females assessed from 3 to 25 postnatal days (dpn).
(DOC)
