# Supplementary material for: Gender Differences in Transcriptional Signature of Developing Rat Testes and Ovaries following Embryonic Exposure to 2,3,7,8-TCDD
Source: PLoS One. 2012 Jul 9;7(7):e40306. doi: 10.1371/journal.pone.0040306 (PMC3392256; doi:10.1371/journal.pone.0040306)
Supplement: Supporting Information S1 — Reproductive parameters of the female progeny exposed in utero to TCDD. (a) F1 female progeny weight from 5 to14 postnatal days. Values (g) are mean ± SEM of () number of pups. TCDD-200 ng females were significantly lighter than control females at 4, 7 and 10 postnatal days (* p<0.05). (b) F1 female fertility assessment. Control females (7) and TCDD-200 ng females (5) were mated continuously with males from 2 to 7 months of age. A total of 5 litters was obtained for each female. Newborn pups were sacrificed after 2 days to check viability. For each litter, the mean number of days between beginning of mating and parturition is indicated for the 7 control and 5 TCDD-treated females. We also recorded the mean number of pups of each sex for the 7 control and 5 TCDD-treated females. (c) Expression levels of key genes involved in endocrine function of the ovary. No significant differences were observed between control and TCDD-200 ng treated females assessed through a transcriptomic analysis on 14 dpn ovaries. Values are the mean of the 3 samples analysed by microarray. (d) Real-time RT-PCR measurement of Cyp19a1 and Star genes in ovaries during prepubertal period. Values were normalized using Hprt and are mean ± SEM of () number of ovaries. No significant differences were observed between control and TCDD- 200 ng treated females assessed from 3 to 25 postnatal days (dpn). (DOC) [file pone.0040306.s001.doc]

**SUPPORTING Information S1**

**(a) F1 female progeny weight from 5 to14 postnatal days**

| Age in days | 2, 3, 7, 8 TCDD (ng/kg) | |
| --- | --- | --- |
|  | 0 | 200 |
| 5 | 13.33 + 0.58 (18) | 11.79 + 0.4 (24)* |
|  |  |  |
| 7 | 18.71 + 0.81 (14) | 16.22 + 0.55 (20)* |
|  |  |  |
| 10 | 26.76 + 1.12 (14) | 23.68 + 0.82 (20)* |
|  |  |  |
| 12 | 31.8 + 1.61 (10) | 29.38 + 1.18 (15) |
|  |  |  |
| 14 | 39.5 + 2.44 (6) | 35.24 + 1.83 (9) |
|  |  |  |

**(b) F1 female fertility assessment**

|  | days before parturition | | number of males | | number of females | |
| --- | --- | --- | --- | --- | --- | --- |
|  | controls | TCDD | controls | TCDD | controls | TCDD |
| Litter 1 | 27 | 34.4 | 6.4 | 6.2 | 7.29 | 6 |
| Litter 2 | 26 | 27.8 | 6 | 6.4 | 6.43 | 6.2 |
| Litter 3 | 25.9 | 26.8 | 4.71 | 6.25 | 6.71 | 6.5 |
| Litter 4 | 27.9 | 27.4 | 6.14 | 7.2 | 7.29 | 5.8 |
| Litter 5 | 29 | 24.6 | 5.83 | 6.4 | 5.5 | 6.6 |

**(c) Expression levels of key genes involved in endocrine function of the ovary**

| **probeset ID** | **genes** | **control** | **TCDD** | ***p* value** |
| --- | --- | --- | --- | --- |
|  |  |  |  |  |
| 1369444_at | Cyp19a1 | 275 | 402 | 0.09 |
|  |  |  |  |  |
| 1368406_at | Star | 4285 | 4122 | 0.94 |
|  |  |  |  |  |
| 1369033_at | LH receptor | 86 | 87 | 0.96 |
|  |  |  |  |  |
| 1368468_at | Cyp11a1 | 5693 | 7277 | 0.11 |
|  |  |  |  |  |
| 1387123_at | Cyp17a1 | 16531 | 16463 | 0.84 |
|  |  |  |  |  |

**(d) Real-time RT-PCR measurement of Cyp19a1 and Star genes in ovaries during prepubertal period**

|  | **Cyp19a1** | | **Star** | |
| --- | --- | --- | --- | --- |
|  | control | TCDD | control | TCDD |
| 3 dpn | 0.11 + 0.01(2) | 0.23 + 0.02(4) | 0.13 + 0.03(2) | 0.17 + 0.03(4) |
| 6 dpn | 1.96 + 0.23(4) | 1.07 + 0.48(3) | 0.55 + 0.09(4) | 0.34 + 0.09(3) |
| 10 dpn | 1.54 + 0.45(4) | 1.87 + 0.78(5) | 1.13 + 0.59(4) | 0.85 + 0.16(5) |
| 12 dpn | 1.19 + 0.32(4) | 1.27 + 0.49(6) | 1.82 + 0.67(4) | 1.20 + 0.28(6) |
| 14 dpn | 0.50 + 0.15(4) | 0.90 + 0.15(5) | 1.45 + 0.77(4) | 1.63 + 0.21(5) |
| 25 dpn | 0.75 + 0.26(2) | 0.76 + 0.26(4) | 0.93 + 0.51(2) | 1.12 + 0.18(4) |
